# Plasma Levels of Organochlorine Pesticides and Tumor Necrosis Factor-Alpha: A Potential Risk Factor for Developing Acquired Aplastic Anemia in the North Indian Population

**DOI:** 10.7759/cureus.46122

**Published:** 2023-09-28

**Authors:** Charu Goel, Nidhish Kumar, Anil K Tripathi, Sunita Tiwari, Ashutosh Shrivastava, Saurabh Shukla, Alok Mishra, Anshuman Srivastava

**Affiliations:** 1 Physiology, Muzaffarnagar Medical College & Hospital, Muzaffarnagar, IND; 2 Physiology, King George's Medical University, Lucknow, IND; 3 Pathology, Autonomous State Medical College, Shahjahanpur, IND; 4 Clinical Hematology, King George's Medical University, Lucknow, IND; 5 Physiology, Dr. Ram Manohar Lohia Institute of Medical Sciences, Lucknow, IND; 6 Centre for Advanced Research, King George's Medical University, Lucknow, IND

**Keywords:** heptachlor, tumor necrosis factor-alpha (tnfα), delta-hch, mini quechers, gc-ms/ms, pesticide, organochlorine, aplastic anemia

## Abstract

Background

Pesticide exposure might have a contributory role in the development of acquired aplastic anemia (AA). However, the precise mechanisms of pesticide-induced AA remain unknown. In this case-control study, we conducted a comparative analysis of plasma levels of organochlorine pesticides (OCP) and tumor necrosis factor-alpha (TNF-alpha) between Indian patients diagnosed with AA and an age- and sex-matched control group.

Methods

This is an observational case-control study conducted at a tertiary care hospital in North India. In this study, 90 subjects were included, out of which 45 were diagnosed with AA according to the criteria of the International Agranulocytosis and Aplastic Anemia Study. Cases were compared with 45 controls. A trained interviewer gave all study subjects a questionnaire to collect data regarding demographic details, exposure to pesticides, and clinical history. Physical examination and routine laboratory investigations of each subject were performed. Both cases and controls were tested for their plasma levels of organochlorines as per established protocol by gas chromatography-mass spectrometry. TNF-alpha level was measured by enzyme-linked immunosorbent assay in each subject.

Results

There was a significant increase in plasma levels of delta hexachlorocyclohexane (delta HCH) (p = 0.02) and heptachlor (p = 0.00) in patients with AA as compared to controls. We observed nonsignificant trends towards higher levels of beta HCH (p = 0.643), aldrin (p = 0.399), and p,p’-Dichlorodiphenyltrichloroethane (p,p’-DDT) (p = 0.453) in patients with AA when compared to the controls. There were significantly higher TNF-alpha levels (p = 0.024) in cases as compared to the controls.

Conclusion

Our study concludes that patients with AA exhibited higher levels of delta-HCH, heptachlor, and TNF-alpha in comparison to the control group. There is a significant positive correlation of TNF alpha with OCPs (alpha HCH, lindane, delta HCH, heptachlor, aldrin, p,p’- DDD, and methoxychlor pesticides). These organochlorines may have accumulated in the fatty tissue of bone marrow because of their lipophilic nature. This suggests that they might have served as a neoantigen to trigger an increase in TNF-alpha production, which may have led to disrupted bone marrow function through cell-mediated immunity, leading to AA.

## Introduction

Aplastic anemia (AA) affects two to seven million individuals globally [1]. It is a disease characterized by a lack of red blood cells, neutrophils, monocytes, platelets in the blood, and hematopoietic stem cells replaced by fatty tissue [2]. People in Asian nations are two to three times more impacted than those in other regions [3]. While the prevalence of AA is significantly higher in developing nations than in industrialized countries, there is little evidence that pesticide exposure is the core cause of idiopathic AA.

AA’s development has been linked to pesticide exposure, including organophosphate chemicals and chlorinated hydrocarbons [4]. Dichlorodiphenyltrichloroethane (DDT), lindane, and chlordane are the most common insecticides involved. Lindane is partially degraded to pentachlorophenol (PCP), a potentially harmful chlorinated hydrocarbons used as a wood preservative. For the past 25 years, PCP has been linked to many occurrences of AA and other blood diseases [5]. Hence, diminished hematopoiesis appears to be the result of acquired toxic effects on primitive hematopoietic cells or, alternatively, immunological suppression of hematopoietic progenitor cells. The data suggest autoreactive T cells decrease hematopoiesis [6].

In India, the prevalence of AA remains uncertain due to limited epidemiological research. However, a study conducted by Mahapatra et al. analyzed 1501 patients over a 7.5-year period (January 2007-June 2014). The study revealed that the majority of patients originated from Uttar Pradesh (28.7%), followed by Bihar (23.6%), the Delhi region (20%), and Haryana (7%) [7]. Another study by Ahamed et al. (2006) estimated an incidence rate of 6.8 cases of pediatric AA per million individuals in India [8]. In contrast to Japan, where idiopathic cases account for approximately 90% of AA cases, European countries and the United States have a higher percentage (40%-70%) of cases with an unknown etiology [9]. Rugman and Costick’s study shows three individuals with AA who have a history of prior exposure to organochlorine pesticides (OCP) [6].

Case reports show a significant association between OCP exposure and the subsequent development of AA [5]. Pesticide exposure leads to blood residue accumulation, immune system modulation, and oxidative damage, with more significant alterations in insecticide-exposed workers compared to fungicide-exposed workers [10]. The findings of several studies show that those exposed to pesticides who have experienced AA have fueled concerns about the development of AA as a result of pesticide exposure [11,12,5,8]. This study investigated the relationship between AA in the North Indian population and chronic OCP exposure.

## Materials and methods

This case-control study included 90 subjects during the period from May 2018 to November 2019, attending the outpatient department of clinical hematology at a tertiary care hospital in North India. Among these subjects, 45 were diagnosed cases of AA according to established criteria of the International Agranulocytosis and Aplastic Anemia Study [13]. These cases were compared to 45 controls. Controls were age- and sex-matched apparently healthy subjects from the same geographical area and preferably relatives of cases. Subjects not willing to give consent for the study; receiving drugs for the treatment of AA; having a genetic predisposition for AA; subjects with features like dysplasia, granuloma, or another systemic disease; or with a history of smoking, alcohol intake, tobacco consumption in any form were excluded from the study. The study was approved by the Institutional Ethical Committee. Written informed consent was taken from each subject prior to the conduction of the study. Both cases and controls were tested for their plasma levels of OCPs. Organochlorine analysis was done as per the established protocol by gas chromatography-tandem mass spectrometry (GC-MS/MS). Tumor necrosis factor-alpha (TNF-alpha) level was measured by enzyme-linked immunosorbent assay using Krishgen Biosystems Human Elisa kit (Krishgen Biosystems, CA). All study groups were given a questionnaire by a trained interviewer, and subsequently, data were collected. Prior to the study’s execution, each participant underwent a thorough medical examination. The sample collection was performed before the start of any treatment.

Sample collection

The blood samples were collected and centrifuged at around 3000 rpm for 15 minutes in a centrifuge machine. Plasma was collected with care to avoid hemolysis and stored in aliquots at minus 80°C in a deep freezer until use. From the 5 mL blood sample, a 3 mL blood sample was gathered in pre-heparinized vials devoid of pesticides as coded samples, which were then delivered under ice-cold conditions for pesticide analysis. The remaining 2mL was used for hematological and biochemical estimation.

Reagents and solutions

Every chemical and solvent utilized in this investigation was of the analytical grade. The following were procured: acetonitrile and ethyl acetate (Reliable Scientific, Lucknow, India); n-hexane, acetone, and acetic acid (Rankem); magnesium sulphate (MgSO_4_) and sodium chloride (Sigma-Aldrich, Bangalore India); primary-secondary amine (PSA), C18 solid phases (Agilent Technologies); standard for 20 OCPs (Sigma Aldrich). Standard has 20 pesticides (alpha-hexachlorocyclohexane (HCH), beta-HCH, lindane, delta-HCH, heptachlor, aldrin, heptachlor epoxide, endosulfanpeak 1, chlordanealphacis, chlordanegammatrans, p,p’- DDE, dieldrin, endrin, endosulfanpeak 2, p,p’- DDD, endrin aldehyde, p,p’- DDT, endosulfan sulfate, endrinKetone, and methoxychlor).

GC-MS/MS analysis

GC-MS/MS analysis was carried out at the molecular biology lab of the Centre for Advanced Research. Organochlorine analysis was done as per the established protocol by a mini QuEChERS methodology used in the GCMS/MS [14].

Pesticide residue extraction and cleanup

A sample of 1 mL of plasma was taken in a 15-ml polypropylene centrifuge tube and 3 ml of 2% ethyl acetate (acidified; concentrated acetic acid was used to acidify ethyl acetate) was added and mixed into it. Then 0.4 gm of MgSO_4_ was added, and the tube was shaken for 5 minutes at 50 rpm on a Rotospin rotary mixer and centrifuged for 10 minutes at 6000 rpm. Using a Turbovap nitrogen flow evaporator, a 3-ml aliquot of the organic layer was separated and evaporated to dryness. For sample cleaning, the residue was reconstituted in 1 ml of ethyl acetate and mixed with 50 mg of PSA. The mixture was shaken for 5 minutes at 50 rpm using a Rotospin rotary and was centrifuged at 8000 rpm for 10 minutes. With 100 μl of ethyl acetate, the residue was reconstituted. For further analysis, 2 μl was injected into the GC-MS/MS apparatus. GC-MS/MS analytical conditions were maintained, and the analysis was done as per the protocol [14].

Statistical analysis

In the first step of data analysis, the categorical data were presented in the form of frequency and percentage, and continuous and normal distributed variables were presented in the form of mean ± SD. The Kolmogorov-Smirnov test was used to determine the normality of the data. Non-normal data were presented in the form of a median with an interquartile range (IQR). Comparisons between normal and non-normal distributed data were done using independent t-test/ANOVA. Mann-Whitney U and Kruskal-Wallis tests were used for the comparison of normal and non-normal data.

Using Statistical Package for the Social Sciences (IBM SPSS Statistics for Windows, IBM Corp., Version 28.0, Armonk, NY), the data were analyzed. Statistical significance was established as a two-tailed p-value to be 0.05.

## Results

There was no significant difference in age as well as sex between cases and controls, as shown in Table 1. Out of the total 45 cases, there were 30 males and 15 females with a mean age of 36.59 ± 13.79 years, whereas out of 45 controls, there were 34 males and 11 females with a mean age of 40.38 ± 13.36 years.

**Table 1:** Age and sex distribution among AA patients and control subjects *p value < 0.05; ***p value < 0.001 AA: aplastic anemia

Characteristics	Study Group (n = 45)	Control Group (n = 45)	T-value/z-value/U test	P-value
Age (Years)	36.59 ± 13.79	40.38 ± 13.36	-1.316	0.192
Sex				
Male	30 (66.7)	34 (75.6)	-0.930	0.352
Female	15 (33.3)	11 (24.4)	0.931	0.351

Table 2 shows there was a significant increase in blood plasma levels of delta HCH and heptachlor and a non-significant increase in beta HCH, aldrin, and p,p’-DDT organochlorine levels in cases as compared to controls, whereas remaining OCPs were having nonsignificant differences in cases vs. controls. In both groups, eight of the pesticides were undetectable. Alpha HCH, lindane, p,p’-DDE, and p,p’-DDD were non-significantly high in controls, whereas endrin-aldehyde,endosulfan-sulfate, and methoxychlor show no difference among cases and controls.

**Table 2:** Organochlorine pesticide levels in blood samples of cases vs. controls (ppb) N/D = not detected; *p - value < 0.05 considered to be significant; HCH - Hexachlorocyclohexane; p,p’-DDE - p,p'-Dichlorodiphenyl dichloroethylene; p,p’-DDD - p,p'-Dichlorodiphenyl dichloroethane; p,p’-DDT - p,p’-Dichlorodiphenyl trichloroethane

S. No	Organochlorine Pesticide	Study Group (n = 45; ppb)	Control Group (n = 45; ppb)	Mann-Whitney U test	P-value
1	Alpha HCH	0.386 (0.321, 0.433)	0.416 (0.332, 0.539)	858.00	0.121
2	Beta HCH	1.049 (0.425, 2.395)	0.811 (0.465, 2.342)	955.00	0.643
3	Lindane	0.40 7(0.351, 0.512)	0.422 (0.338, 0.894)	963.00	0.690
4	Delta HCH	1.162 (1.033, 1.296)	1.005 (0.895, 1.215)	639.00	0.002*
5	Heptachlor	0.58 7(0.438, 1.598)	0.0007 (0.0007, 0.5094)	299.00	0.000*
6	Aldrin	44.870 (20.962, 57.808)	35.147 (1.336, 62.687)	804.50	0.399
7	Heptachlorepoxide	N/D	N/D	0	0
8	Chlordanealphacis	N/D	N/D	0	0
9	Endosulfanpeak1	N/D	N/D	0	0
10	Chlordanegammatrans	N/D	N/D	0	0
11	p,p’-DDE	6.908 (3.142, 14.073)	9.423 (3.993, 20.748)	723.00	0.119
12	Dieldrin	N/D	N/D	0	0
13	Endrin	N/D	N/D	0	0
14	Endosulfanpeak2	N/D	N/D	0	0
15	p,p’-DDD	0.542 (0.121, 1.21)	0.629 (0.121, 1.245)	893.00	0.949
16	Endrin Aldehyde	0.172 (0.172, 0.172)	0.172 (0.172, 0.172)	852.00	0.504
17	p,p’-DDT	4.609 (1.594, 11.739)	4.204 (0.995, 7.930)	815.00	0.453
18	Endosulfansulfate	1.049 (1.049, 1.049)	1.049 (1.049, 1.049)	820.00	0.065
19	EndrinKetone	N/D	N/D	0	0
20	Methoxychlor	0.914 (0.914, 0.914)	0.914 (0.914, 0.914)	821.00	0.193

In this study, the TNF-alpha level was 13.22 (0.891, 46.619) pg/ml and was significantly high (p = 0.024) in cases compared to controls at 7.86 (3.000, 27.285) pg/ml.

In Table 3 correlation between variables was done using Spearman Rank Correlation. There was a significant positive correlation of TNF alpha with alpha HCH, lindane, delta HCH, heptachlor, aldrin, p,p’-DDD, and methoxychlor pesticides while p,p’-DDE, endrin aldehyde, p,p’-DDT, show non-significant positive correlation. Where as in controls TNF alpha was correlated non-significantly and positively to all the pesticides except beta HCH.

**Table 3:** Correlation of TNF-alpha with the organochlorine pesticides in cases and controls *Correlation is significant at the 0.05 level (2-tailed). ** Correlation is significant at the 0.01 level (2-tailed).

		TNF-alpha (Cases)		TNF-alpha (Controls)	
		Correlation Coefficient	Sig. (2-tailed)	Correlation Coefficient	Sig. (2-tailed)
Alpha HCH		0.383^*^	0.01	0.039	0.809
Beta HCH		-0.155	0.315	-0.104	0.524
Lindane		0.419^**^	0.005	0.16	0.324
Delta HCH		0.328^*^	0.03	0.07	0.668
Heptachlor		0.482^**^	0.001	0.122	0.454
Aldrin		0.594^**^	0	0.1	0.633
p,p’-DDE		0.197	0.199	0.043	0.791
p,p’-DDD		0.544^**^	0	0.138	0.395
Endrin	Aldehyde	0.091	0.557	0.026	0.875
p,p’-DDT		0.119	0.44	0.092	0.571
Methoxychlor		0.356^*^	0.019	-0.281	0.079

## Discussion

The present study’s findings showed that individuals with AA (case group) had significantly raised plasma levels of the organochlorines delta-HCH (1.162 ppb) and heptachlor (0.587 ppb) while non-significantly raised levels of beta-HCH (1.049 ppb), aldrin (44.870 ppb), and p,p’-DDT (4.609 ppb) compared to the control group, among the studied 20 OCPs. Out of these, eight pesticides remain undetected. Alpha HCH (0.386 ppb), lindane (0.407 ppb), p,p’-DDE (6.908 ppb), and p,p’-DDD (0.542 ppb) were non-significantly high in controls, whereas endrin-aldehyde, endosulfan-sulfate, and methoxychlor show no significant difference among cases as compared to controls. It was seen in our study that there is a significant positive correlation of TNF alpha with alpha HCH, lindane, delta HCH, heptachlor, aldrin, p,p’-DDD, and methoxychlor pesticides while p,p’-DDE, endrin aldehyde, p,p’-DDT, show a non-significant positive correlation. Whereas in controls TNF alpha was correlated non-significantly and positively to all the pesticides except beta HCH. This shows organochlorines may play a role in the development of AA. Our study results are consistent with the previous studies [15].

A review of various case reports from 1946 to 1960 shows that exposure to organochlorines particularly can produce blood dyscrasias. However, it is unclear whether this relationship serves as a causal factor [16]. Studies show that OCPs can affect the hematopoietic system through immunological mechanisms, causing blood-related disorders [17]. Because fat is a key component in the stromal support of hematopoiesis, bioaccumulation of OCPs in bone marrow adipose tissue may increase the risk of interaction with lymphohematopoietic function [18]. Rugman and Costick have explained that the organochlorines or their metabolites may become immunogenic by interacting with a certain uncommon, undiscovered human leukocyte antigen molecule, leading to an autoimmune reaction that causes bone marrow destruction in a continuous fashion. Another research study demonstrates that fat plays a crucial role in the stromal support of hemopoiesis [19], and as a result, the likelihood of an interaction between OCPs and lymphohematopoietic function may be increased if they are known to persist in bone marrow adipose issue. For instance, phytohemagglutinin-stimulated lymphocyte proliferation is inhibited by lindane [20].

In healthy people, even eating meals with pesticide residues may dramatically impair bone marrow function according to research from Israel [21]. Case history by Sanchez-Medal et al. revealed AA-related deaths of hospitalized patients who had reported chronic DDT exposure [22]. In India, technical grade HCH is being replaced with lindane, but DDT is still utilized in malaria prevention programs, thus imposing the risk on many. Our study is consistent with previous studies, showing organochlorines like lindane (gamma-HCH), chlordane, and pentachlorophenol are associated with AA [5,23]. In a study conducted in India, pesticide applicators had hematological abnormalities such as leukopenia, lymphocytopenia, neutropenia, monocytopenia, anemia, and thrombocytopenia compared to the non-exposed group. This association between hematological abnormalities and pesticide exposure was supported by the presence of OC pesticides in blood [24]. Total-HCH and α-HCH residues from organochlorine insecticides were found in the applicator’s blood at considerably greater levels than in the controls’ blood [25].

Patients with AA in our case group have significantly greater plasma levels of TNF-alpha (p = 0.024) compared to the controls. Our findings are in line with earlier work. The plasma concentrations of TNF-alpha and IFN-gamma were found to be considerably greater in patients with AA than those of the controls [26]. Immune-mediated stem cell damage has been related to the onset and development of AA. T-lymphocytes are thought to invade AA patients’ bone marrow, while TNF-alpha and interferon-gamma are anti-hematopoietic cytokines released in large quantities that are crucial in the destruction of bone marrow stem cells. One of the important negative regulators of hematopoiesis is TNF-alpha. Overexpression of TNF-alpha also activates the intracellular death pathway, which results in hematopoietic cell death and failure of bone marrow hematopoiesis in addition to directly suppressing hematopoietic cell proliferation and differentiation [27]. In another study, plasma concentrations of TNF-alpha and IFN-gamma were higher in patients with AA relative to those in controls [28]. TNF-alpha, malondialdehyde, and IL-6 levels were shown to be elevated in the muscles of rats exposed to the pesticide omethoate, which is often used in developing nations, according to a study conducted by Zhang et al. [29].

This study discovered a significant rise in cell-mediated immune biomarker TNF-alpha along with the significantly raised plasma levels of organochlorines among cases as compared to controls. Our study shows a significant positive correlation of TNF-alpha with certain specific OCPs as discussed above. This indicates that hematopoiesis could be disrupted by direct toxicity or indirectly by an immune-mediated mechanism. Organochlorines (delta-HCH and heptachlor) may have accumulated in the fatty tissue of bone marrow because of their lipophilicity, which suggests that they may have served as a neoantigen to trigger an increase in TNF-alpha production, which may disrupt bone marrow function through cell-mediated immunity. However, due to the limitations of the study, a population-based study and prospective follow-up studies are warranted.

## Conclusions

Our study concludes that there is a significant positive correlation of TNF alpha with alpha HCH, lindane, delta HCH, heptachlor, aldrin, p,p’-DDD, and methoxychlor pesticides. These organochlorines may have accumulated in the fatty tissue of bone marrow because of their lipophilicity. This suggests that they could have served as a neoantigen to trigger an increase in TNF-alpha production, which could have led to disrupted bone marrow function through cell-mediated immunity, leading to AA.

The results of the current study help in understanding of potential risks of developing AA against a backdrop of environmental exposures to organochlorines. Additional long-term studies are necessary to determine whether one of these agents or a combination of these agents along with other groups of pesticides is to blame for this in order to confirm this link. Our results indicate the need for a population-based study and a prospective follow-up study in this regard.
